# Association of Cancer Stem Cell Radio-Resistance Under Ultra-High Dose Rate FLASH Irradiation With Lysosome-Mediated Autophagy

**DOI:** 10.3389/fcell.2021.672693

**Published:** 2021-04-29

**Authors:** Gen Yang, Chunyang Lu, Zhusong Mei, Xiaoyi Sun, Jintao Han, Jing Qian, Yulan Liang, Zhuo Pan, Defeng Kong, Shirui Xu, Zhipeng Liu, Ying Gao, Guijun Qi, Yinren Shou, Shiyou Chen, Zhengxuan Cao, Ye Zhao, Chen Lin, Yanying Zhao, Yixing Geng, Wenjun Ma, Xueqing Yan

**Affiliations:** ^1^State Key Laboratory of Nuclear Physics and Technology, School of Physics, CAPT, Peking University, Beijing, China; ^2^Teaching and Research Section of Nuclear Medicine, School of Basic Medical Sciences, Anhui Medical University, Hefei, China; ^3^Collaborative Innovation Center of Extreme Optics, Shanxi University, Taiyuan, China

**Keywords:** ultra-high dose rate irradiation, FLASH, DNA damage, lysosome, cancer stem cell, autophagy, apoptosis, necrosis

## Abstract

Cancer stem cell (CSC) is thought to be the major cause of radio-resistance and relapse post radiotherapy (RT). Recently ultra-high dose rate “FLASH-RT” evokes great interest for its decreasing normal tissue damages while maintaining tumor responses compared with conventional dose rate RT. However, the killing effect and mechanism of FLASH irradiation (FLASH-IR) on CSC and normal cancer cell are still unclear. Presently the radiation induced death profile of CSC and normal cancer cell were studied. Cells were irradiated with FLASH-IR (∼10^9^ Gy/s) at the dose of 6–9 Gy via laser-accelerated nanosecond particles. Then the ratio of apoptosis, pyroptosis and necrosis were determined. The results showed that FLASH-IR can induce apoptosis, pyroptosis and necrosis in both CSC and normal cancer cell with different ratios. And CSC was more resistant to radiation than normal cancer cell under FLASH-IR. Further experiments tracing lysosome and autophagy showed that CSCs had higher levels of lysosome and autophagy. Taken together, our results suggested that the radio-resistance of CSC may associate with the increase of lysosome-mediated autophagy, and the decrease of apoptosis, necrosis and pyroptosis. To our limited knowledge, this is the first report shedding light on the killing effects and death pathways of CSC and normal cancer cell under FLASH-IR. By clarifying the death pathways of CSC and normal cancer cell under FLASH-IR, it may help us improve the understanding of the radio-resistance of CSC and thus help to optimize the future clinical FLASH treatment plan.

## Introduction

Cancer is one of the leading causes of death, more than half of cancer patients receive radiotherapy (RT) during treatment (Arnold et al., 2020). In RT, the common types of radiation are photon (such as X-rays and γ-rays) radiation and particle (such as electrons, protons, heavy ions and neutrons) radiation. RT exerts cell killing effect by inducing DNA damage, either directly or indirectly via generating free radicals by interacting with water molecules (YuN, 1992). Irradiation induces cell death mainly by damaging DNA. Among them, apoptosis and necrosis are very important death pathways (Mitteer et al., 2015; Bozhenko et al., 2019; Wang et al., 2019). Besides, studies have shown that irradiation can also cause cell pyroptosis (Liao et al., 2017; Li et al., 2019). Pyroptosis, as a highly pro-inflammatory programmed cell death, the feature is that it depends on the activation of caspase-1 and releases the inflammatory cytokines IL-1β and IL-18 (Westbom et al., 2015; Wang et al., 2017). In addition, irradiation can also induce cell autophagy. In the early years, autophagy was considered a mechanism by which cells produce nutrients and energy. However, in recent years, many studies have shown when cells were continuously exposed to stress conditions, autophagy acted as a cell stress protection mechanism (Turcotte and Giaccia, 2010), can remove macromolecules or mitochondria that were damaged by ionizing radiation and cytotoxic substances in the cell, and further promote cell survival (Han et al., 2018).

The radio-resistance of cancer stem cells (CSCs) is an important cause of RT failure, cancer recurrence and metastasis (Bao et al., 2006; Chiou et al., 2008; Arnold et al., 2020). Compared with normal cancer cells, the main reasons for CSCs radio-resistance are suggested as follows: (1) CSCs have stronger reactive oxygen species (ROS) removal ability, the ROS baseline in CSCs is lower, and the damage caused by irradiation is less (Diehn et al., 2009; Chang et al., 2014). (2) CSCs have enhanced DNA repair capability (Bao et al., 2006). (3) CSCs are usually in a stationary phase (Moore and Lyle, 2011; Batlle and Clevers, 2017). (4) CSCs have enhanced self-renewal ability (Woodward et al., 2007; Batlle and Clevers, 2017). (5) CSCs have an increased autophagy level (Lomonaco et al., 2009). Among them, in recent years, some studies have shown that lysosomes and autophagy may be related to the radio-resistance of CSC (Tang et al., 2018; Zhang et al., 2018). Through limited activation of autophagy, CSCs can achieve higher cell survival. Blocking autophagy is considered to be a feasible strategy to enhance radio-sensitivity, and autophagy inhibitors are considered as promising radiosensitizers (Zhang et al., 2018).

The ultra-high dose rate FLASH-RT has attracted widely attention in recent years (Bourhis et al., 2019). It greatly protects normal tissues from damage, while effectively killing tumor tissues. This significantly improves the therapeutic index during RT and may achieve better control of the tumor (Durante et al., 2018; Griffin et al., 2020). Compared with conventional dose rate irradiation (∼1 Gy/min), FLASH-RT has a higher dose rate (>40 Gy/s), and the irradiation process is usually completed in a very short time (<0.1 s) (Durante et al., 2018). In the last few decades, important progress on laser plasma acceleration provided a new method to study radiobiology effects (Ledingham et al., 2014). The laser-driven ion beams have ultra-short pulse duration in a range of nanoseconds and ultra-high dose rate in the order of 10^9^ Gy/s (Bin et al., 2012; Doria et al., 2012; Pommarel et al., 2017; Hantonl et al., 2019), showing the potential for *in vitro* (Yogo et al., 2011; Zeil et al., 2013; Raschke et al., 2016; Bayart et al., 2019) and *in vivo* (Rosch et al., 2020) FLASH-RT besides cyclotrons, synchrotrons or synchrocyclotron (Patriarca et al., 2018). Presently, there are few articles on FLASH-RT, mainly because the realization of ultra-high dose rate is quite difficult (Griffin et al., 2020). The existing FLASH-RT articles mainly focus on the protective effect of FLASH on normal tissues (Field and Bewley, 1974; Favaudon et al., 2014; Montay-Gruel et al., 2017; Vozenin et al., 2019). For example, a number of animal FLASH-RT experiments showed that compared with conventional irradiation, FLASH-RT can significantly reduce damage to normal tissues, including reducing pulmonary fibrosis (Favaudon et al., 2014), reducing radiation-induced neurotoxicity (Montay-Gruel et al., 2017) and reducing radiation-induced skin reactions (Field and Bewley, 1974; Vozenin et al., 2019), etc. Besides, there were also several animal experiments proved that FLASH-RT has a tumor-killing effect similar to conventional irradiation (Favaudon et al., 2014; Rama et al., 2019). But there are few researches on the basic killing effect and specific killing mechanisms of FLASH on cancer cells, especially for the important problem of radiation biology, the CSC radio-resistance.

In this article, we used the CLAPA ultra-fast laser acceleration system to irradiate cells with ultra-high dose rate (∼10^9^ Gy/s), and the dose was controlled at 6–9 Gy. The ratio of apoptosis, pyroptosis and necrosis was determined at 6, 12, and 24 h after irradiation. At the same time, we also labeled the lysosomes and detected autophagy in cells.

## Materials and Methods

### Cell Culture

MCF-7 cells were cultured in DMEM high glucose medium (Hyclone) supplemented with 10% fetal bovine serum (FBS) (Bai Ling Biotechnology) and 1% penicillin-streptomycin (P/S) (Hyclone). MCF-7 CSCs were cultured in DME/F12 1:1 medium (Hyclone) supplemented with N2 (Gibco), B27 (Gibco), epidermal growth factor (EGF) (20 ng/mL, PeproTech), and basic fibroblast growth factor (bFGF) (10 ng/m, PeproTech). And the petri dishes used for CSCs were pre-coated with 1% Geltrex (Gibco) as described previously (Fu et al., 2017). All cells were cultured at 37°C under 5% CO_2_ in a humidified incubator (Sanyo).

### Flow Cytometry Analysis

MCF-7 CSCs were obtained by sorting as reported previously (Fu et al., 2017). In brief, the cells were concentrated to a few million/100 μL, and were stained by adding 10 μL anti-CD44-PE antibody (BD Pharmingen) and 10 μL anti-CD24-FITC antibody (BD Pharmingen). After the staining was completed, the cells were washed twice by PBS and analyzed/sorted on flow cytometer (BD Aria III).

### Spheroids Formation Assay

MCF-7 cells and CSCs were digested, centrifuged and resuspended in serum-free medium (CSC culture medium, as mentioned above), the density was adjusted to 2,500 cells/mL, then the cell suspension was added to a low-adsorption 24-well plate (1 mL per well). Cells were cultured at 37°C under 5% CO_2_. After 3 days, spheroids were imaged and with diameter larger than 50 μm were counted. Image acquisition and analysis were performed through automatic real-time live cell imaging detection system (Spark^®^Cyto, Tecan).

### *In vivo* Tumor Formation Experiment in Mice

MCF-7 cells and CSCs were digested, washed three times using PBS, and diluted to a certain density, then mixed the cell suspension and Geltrex (Gibco) at 1:1. Same number (10,000) of MCF-7 cells and CSCs were injected on the left and right fat pads of each BALB/C nude mice, respectively. The growth of the tumor was recorded and the images were token 36 days after injection. All animal experiments and protocols were approved by the Peking University Institutional Animal Care and Use Committee (Approval ID: Physics-YangG-2).

### Implementation of FLASH-IR

The FLASH radiation experiment was performed using CLAPA system in Peking University (Geng et al., 2018). The CLAPA was driven by a 200TW Ti: sapphire laser with central wavelength at 800 nm and full width at half maximum (FWHM) duration of 30 fs compressed. A single plasma mirror system was used to increase the temporal contrast of the laser to 10^8^@5 ps. S-polarized laser was focused onto the 100 nm plastic target using an f/2.5 off-axis parabolic (OAP) with a focal length of 200 mm. The laser energy on target was ∼1 J and the diameter (FWHM) of focal spot was 4.2 μm, containing 36% of the total energy, resulting in the peak intensity of 5.6 × 10^19^ W/cm^2^. The ion energy spectra were measured by a Thomson parabola spectrometer (TPS) positioned at 780 mm along the direction normal to the back surface of the target.

A 3.5 μm Mylar film was pasted at the bottom of the cell-culture hoop using glue in advance, and then was pre-treated with DMEM high glucose medium and/or 1% Geltrex for 24 h, respectively. The height and inner diameter of the stainless-steel cell-culture hoop were 13 and 10 mm, respectively. Cells were seeded 8 h before irradiation. MCF-7 cells and CSCs were digested and diluted to 150,000/mL, and 800 μL cell suspension was added to each cell-culture hoop. Before starting the irradiation, the medium in cell-culture hoop was aspirated, and the cell-culture hoop was screwed on the hoop holder and mounted vertically. The hoop holder was sealed with another 3.5 μm Mylar film to avoid contamination and dryout of the cell during irradiation. Then the cells irradiation system was plugged in, the ion beams entered the cell irradiation system through a vacuum window located 63 mm behind the target and irradiated the cells. The vacuum window consisted of two parts, a 10 μm Aluminum film to prevent cells from being exposed to scattered light and a 25 μm Kapton window for vacuum sealing. Aluminum films of different thicknesses and copper wire mesh were used in front of the sealing Mylar film to adjust the dose. After the irradiation was completed, the medium was added and the cells were inoculated in the incubator.

### Detection of Apoptotic and Necrosis

Dead Cell Apoptosis Kit with Annexin V Alexa Fluor^TM^ 488 & Propidium Iodide (PI) (Invitrogen) were used to detect apoptotic and necrosis. For a 96-well plate, 100 μL cell suspension, 1 μL of PI working solution (red), 5 μL of Annexin V (green) and appropriate volume of Hoechst (blue, Molecular Probes) were added into each well. The images were token after staining according to the manufactural protocol. The nucleus was marked as blue, living cells were blue+, apoptotic cells were blue+green+, and dead cells were marked as blue+green+red+. Image acquisition were performed via automatic real-time live cell imaging detection system (Spark^®^Cyto, Tecan).

### Detection of Pyroptosis

Pyroptosis were detected using Caspase-glo 1 inflammasome assay (Promega), for a 96-well plate, 100 μL of cell suspension and 100 μL Z-WEHD substrate were added, then the chemiluminescence was detected after 1 h. The detection was performed via automatic real-time live cell imaging detection system (Spark^®^Cyto, Tecan).

### Autophagy Detection

After the irradiation is completed, the cells were digested from the Mylar film and seeded into a 96-well plate. Autophagy was detected after 24 h using dansylcadaverine (MDC) autophagy detection kit (Leagene Biotechnology). In addition, lysosomes were labeled with lysoTracker (Invitrogen), nucleus were labeled with Hoechst 33342. Image acquisition was performed via automatic real-time live cell imaging detection system (Spark^®^Cyto, Tecan).

### Data Analysis

All results were expressed as means ± SD, Student’s *t*-test or one-way ANOVA were used for analyzing significance between the controls and the experimental groups, where *P* < 0.05 was marked as ^∗^, *P* < 0.01 was marked as ^∗∗^, and *P* < 0.001 was marked as ^∗∗∗^.

## Results

### Functional Verification of MCF-7 CSCs

**FIGURE 1:**
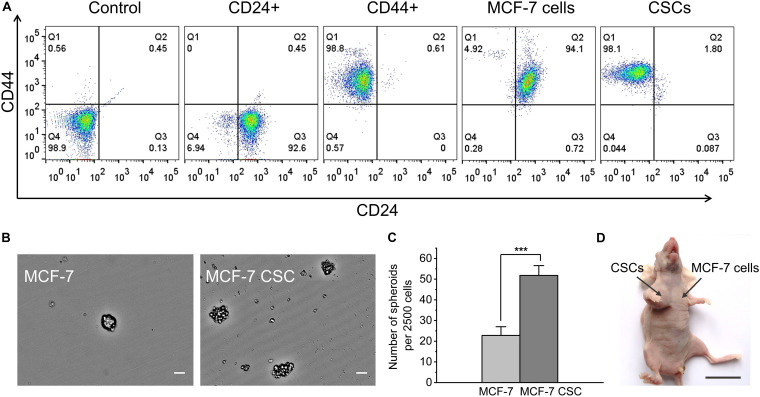
Functional verification of MCF-7 CSCs. **(A)** Flow cytometry results for five groups of cells. From left to right are the control (not stained), the single CD44 stained group, the single CD24 stained group and the CD44CD24 double stained group (MCF-7 cells and CSCs). **(B)** Representative images of spheroids formed by MCF-7 cells and MCF-7 CSCs. Scale bars: 50 μm. **(C)** Quantification of the number of spheroids derived from MCF-7 cells and MCF-7 CSCs at day 3. **(D)** Representative image depicting *in vivo* tumor formation at the injection site of 10,000 CSCs, with no tumor formation seen at the injection site of 10,000 MCF-7 cells. Scale bar: 2 cm. ***P < 0.001.

MCF-7 CSCs were obtained by flow sorting as our research group reported previously (Fu et al., 2017). We used flow cytometry analysis, spheroids formation assay and *in vivo* tumor formation experiment to verify the sorted CSCs. For flow cytometry analysis, the cells were divided into five groups, the control (not stained), the single CD44 stained group, the single CD24 stained group and the CD44CD24 double stained group (MCF-7 cells and CSCs). By analyzing the expression of CD44 and CD24 on the cell surface, the results showed that MCF-7 CSCs maintains the characteristics of CSC, and the percentage of CD44+CD24− cells is 98.1% (Figure 1A). In addition, MCF-7 CSCs also showed stronger self-renewal ability, its spheroids formation efficiency was ∼2.3 folds that of MCF-7 cells (Figures 1B,C). To investigate whether sorted CSCs display long-term tumorigenic potential, we also performed *in vivo* tumor formation experiment by injecting 10,000 MCF-7 cells and CSCs into BALB/C nude mice, and observed and counted the growth of the tumor. The results showed that CSCs have stronger tumorigenesis ability *in vivo*. 10,000 CSCs are capable of generating visible tumors after 36 days (Table 1). In contrast, no visible tumors were observed with MCF-7 cells under the same conditions (Figure 1D). Statistics on the size of tumors were shown in Supplementary Table 1.

**TABLE 1:** *In vivo* tumor formation ability of MCF-7 cells and CSCs.

Cell type	Tumor formation efficiency
MCF-7 cells	0/3
CSCs	2/3

### Dose Monitoring and Conversion of FLASH-IR

During the cell irradiation, customized radiochromic films (RCFs) EBT3 were directly placed behind the Mylar film for dose verification. Since the RCF dose was known, we can get the deposit dose in cells. Considering the fluctuation of the energy spectrum between different shots, the result will have an error of ± 15%. In this experiment, the cells were irradiated with a single shot (∼6–9 Gy). The ion beam was generated within 1 ps. Considering the difference between the flight time of particles with different energy, the duration of radiation was ∼1.4 ns, so the dose rate was ∼10^9^ Gy/s. The specific RCF dose and deposit dose in cells for each shot were shown in Table 2. More detailed information about the dose was shown in Supplementary Figure 1.

**TABLE 2:** Dose summary for each shot of the laser accelerator.

	Dose in RCF film	Deposit dose (Median) in cells	Deposit dose (Range) in cells	Shot used
1	3.05	6.83	5.89–7.88	MCF-7 CSC 6 h
2	3.43	7.68	6.62–8.87	MCF-7 CSC 12 h
3	3.16	7.08	6.10–8.17	MCF-7 CSC 24 h
4	3.93	8.80	7.59–10.16	MCF-7 6 h
5	3.52	7.88	6.80–9.10	MCF-7 12 h
6	3.37	7.54	6.51–8.71	MCF-7 24 h
7	2.82	6.14	5.36–7.07	MCF-7 CSC autophagy
8	3.97	8.89	7.67–10.26	MCF-7 autophagy

### CSCs Are More Resistant Than Normal Cancer Cell to FLASH Irradiation

**FIGURE 2:**
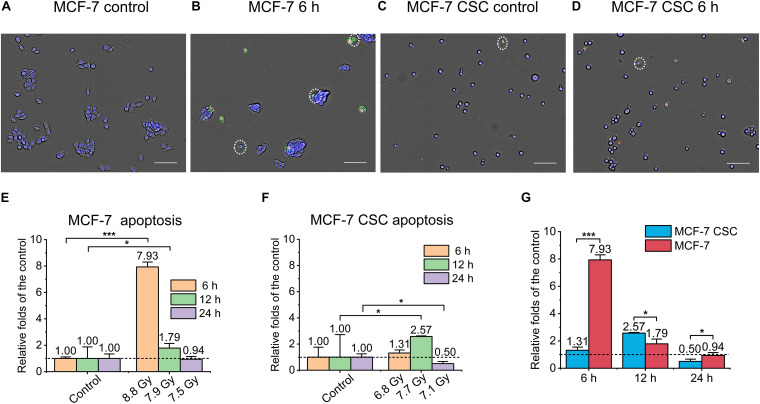
Apoptosis of irradiated MCF-7 cells and MCF-7 CSCs. **(A–D)** Representative images of control groups and irradiated MCF-7 cells and MCF-7 CSCs at 6 h which were stained with annexin V and PI. Apoptotic cells were marked with white circles. Scale bars: 100 μm. **(E,F)** Compared with the relative controls, the relative increasement of the apoptosis proportion for irradiated groups at 6, 12, and 24 h. **(G)** The increasement of the apoptosis proportion between irradiated MCF-7 cells and MCF-7 CSCs. *P < 0.05; ***P < 0.001.

To evaluate the killing effect of ultra-high dose rate FLASH irradiation (FLASH-IR) on MCF-7 and CSC, we assessed the apoptosis, necrosis and pyroptosis, at 6 h, 12 h and 24 h after irradiation. Annexin V Alexa Fluor^TM^x 488 & Propidium Iodide (PI) was used to detect apoptosis and necrosis. Apoptotic cells were marked as blue+green+, and dead cells were marked as blue+green+red+. Figures 2A–D were the representative images showing the differences between MCF-7 and CSC after irradiation, compared with the control, the apoptosis level of irradiated MCF-7 at 6 h was significantly increased, which was ∼7.9 folds that of the control. In addition, there was also an increasement at 12 h, which was ∼1.8 folds of the control (Figure 2E). For CSC, apoptosis had a significant but much lower increasement at 12 h, which was ∼2.6 folds of relative control, while there was no significant change at any other time points (Figure 2F). Putting the apoptosis data of irradiated MCF-7 and CSC together, it can also be seen that the apoptosis level of irradiated MCF-7 was significantly higher than that of irradiated CSC, among them, MCF-7 had the highest response at 6 h after irradiation, while CSC had the highest response at 12 h after irradiation (Figure 2G).

**FIGURE 3:**
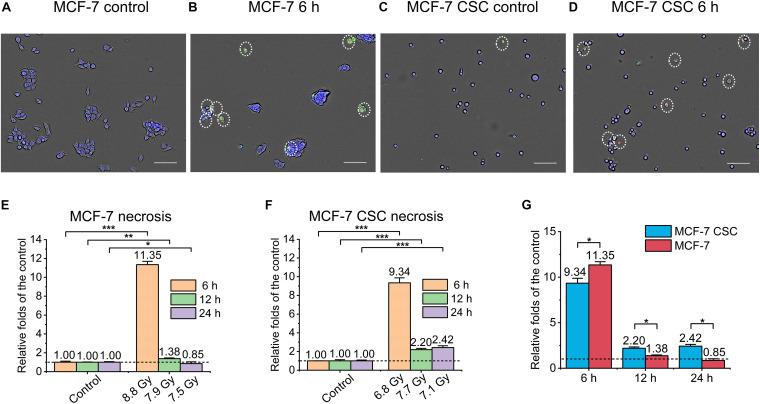
Necrosis of irradiated MCF-7 cells and MCF-7 CSCs. **(A–D)** Representative images of control groups, irradiated MCF-7 cells and MCF-7 CSCs at 6 h which were stained with annexin V and PI. Necrosis cells were marked with white circles. Scale bars: 100 μm. **(E,F)** Compared with the relative controls, the increasement of the necrosis proportion for irradiated groups at 6, 12, and 24 h. **(G)** The increasement of the necrosis proportion between irradiated MCF-7 cells and MCF-7 CSCs. *P < 0.05; **P < 0.01; ***P < 0.001.

Similarly, as shown in Figure 3, we also detected and analyzed the necrosis of the irradiated cells. Figures 3A–D were the representative images. It can be seen that, similar to the result of apoptosis, the necrosis of MCF-7 at 6 h after irradiation was ∼11 folds than that of the control (Figure 3E). On the other hand, the necrosis of CSC at 6 h after irradiation reached ∼9.3 folds than that of the control. In addition, CSC’s necrosis at 12 and 24 h after irradiation also reached ∼2.3 folds that of the control (Figure 3F). Comparing the necrosis of irradiated CSC and MCF-7, the results showed that although the necrosis of CSC in the early stage was lower than MCF-7, in the late stage, it was significant higher (Figure 3G).

**FIGURE 4:**
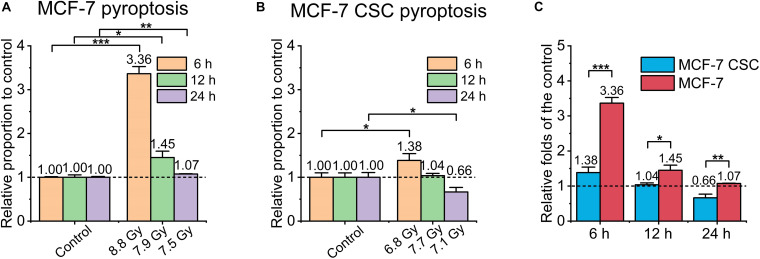
Pyroptosis of irradiated MCF-7 cells and MCF-7 CSCs. **(A,B)** Compared with the relative controls, the increasement of pyroptosis proportion for irradiated groups at 6, 12, and 24 h, respectively. **(C)** The increasement of the pyroptosis proportion between irradiated MCF-7 cells and MCF-7 CSCs. *P < 0.05; **P < 0.01; ***P < 0.001.

Pyroptosis is a highly pro-inflammatory programmed cell death. We made a rough assessment of pyroptosis by detecting the activity of caspase-1 in the cell. Mainly using Caspase-glo 1 inflammasome assay kit. The main principle of the kit is as follows, a single addition of this reagent results in cell lysis, substrate cleavage by caspase-1 and generation of light by a proprietary, thermostable, recombinant luciferase. The luminescent signal is proportional to caspase activity (O’Brien et al., 2017). As shown in Figures 4A–C, the overall normalized pyroptosis level of irradiated MCF-7 was also higher than that of irradiated CSC, the former reached ∼3.4 folds and ∼1.5 folds of the control at 6 and 12 h, respectively, while the latter only reached ∼1.4 folds of the relative control at 6 h, no significant change at any other time points.

### CSCs Increase Autophagy

**FIGURE 5:**
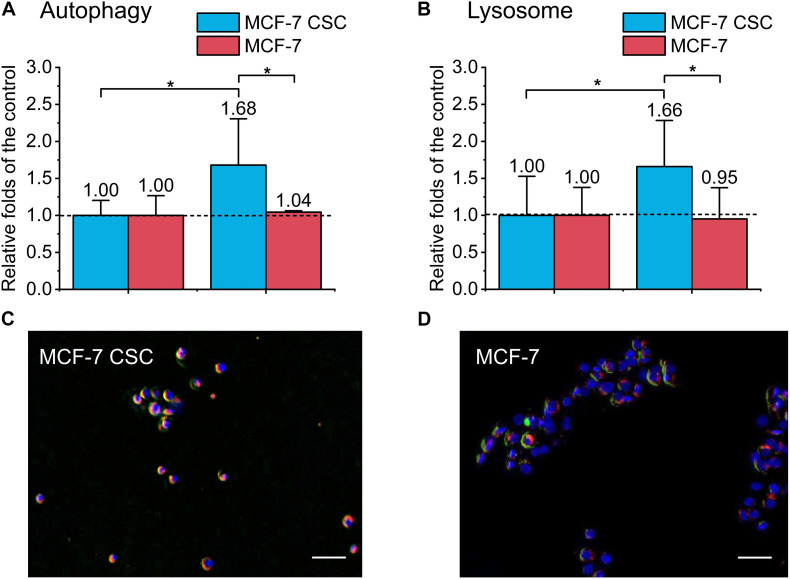
Detection of autophagy and lysosome of irradiated MCF-7 cells and MCF-7 CSCs. **(A)** Compared with the control, the increasement of autophagy proportion for irradiated group. **(B)** Compared with the control, the increasement of lysosome for irradiated group. **(C,D)** Representative images of irradiated MCF-7 cells and MCF-7 CSCs stained with MDC autophagy detection kit and lysoTracker Red DND-99. The blue was the nucleus, the red was the lysosome, and the green labeled autophagy. Scale bars: 50 μm. *P < 0.05.

To further study the mechanism of CSCs’ radio-resistance under FLASH-IR, we used MDC staining kit to detect the autophagy level of the two cell types. We labeled lysosomes and detected autophagy at 24 h after irradiation. The results showed that the autophagy of irradiated CSCs increased significantly compared with the control, which was ∼1.7 folds than that of the control, while the autophagy of irradiated normal cancer cell (MCF-7) almost showed no change compared to the relative control (Figures 5A,B). Figures 5C,D were the representative images.

Overall, our results showed that even under ultra-high dose rate FLASH-IR, MCF-7 CSC was more radio-resistant than normal cancer cells (MCF-7), the proportion of apoptosis was significantly lower. By detecting cell’s autophagy after irradiation, we found that the autophagy in CSC was much higher, which may make it more resistant to radiation and appear less apoptosis. In addition, our experiments also found that the overall level of irradiated pyroptosis and necrosis of CSC was slightly lower than that of normal cells, and the response to irradiation also seemed slower.

## Discussion

In this article, we studied the killing effect of ultra-high dose rate FLASH-IR on MCF-7 and its CSCs. The results showed that the apoptosis of irradiated CSCs was significantly lower than that of MCF-7, and the pyroptosis and necrosis of irradiated CSCs were also slightly lower than that of MCF-7. In addition, the response of CSC to irradiation was slower. These results suggested the radio-resistance of CSCs under FLASH-IR. By detecting cell’s autophagy as well as lysosomes after irradiation, we found that the lysosomes and autophagy in CSC are much higher, suggesting CSC may enhance the radio-resistance by increasing lysosome-mediated autophagy and decreasing apoptosis after FLASH-IR.

High levels of anti-apoptotic molecules make cancer cells resistant to RT and chemotherapy (Schmitt, 2003). Drugs targeting the apoptotic pathway have anticancer effects in many malignant tumors, and many of these new therapies have entered clinical trials (Hassan et al., 2014; Delbridge et al., 2016). Among them, CSCs seemed to be more resistant to apoptosis than normal cancer cells. Bao et al. (2006) reported that the apoptosis in CSCs was ∼4–5 folds lower than that in non-stem cancer cells (NSCCs), in addition, there were also results showing that the apoptosis of CSCs was ∼8 folds lower than that of NSCCs when irradiated by conventional dose rate γ-rays, and was ∼3–4 folds lower than that of NSCCs when irradiated by conventional dose rate proton (Fu et al., 2012). Present experimental results showed that the apoptosis of FLASH irradiated CSCs was ∼6 folds lower than that of irradiated MCF-7 cells, suggesting even under ultra-high dose rate irradiation, the radio-resistance of CSC still existed. The possible reasons for CSCs’ apoptosis resistance are as follows, the critical step in the apoptotic cascade is mitochondrial outer membrane permeabilization (MOMP), and CSCs expressed higher levels of anti-apoptotic proteins related to MOMP, including Bcl-2 and Bcl-XL (Beroukhim et al., 2010). Our results indicated that CSCs produce less pyroptosis than normal cancer cells, which may be related to CSCs’ stronger ROS removal ability (Chang et al., 2014). Ion irradiation induced the production of ROS, and at the same time, the excessive ROS destroyed the body’s antioxidant system, thereby generating more ROS, forming a cascade of amplified inflammatory responses (Qiu et al., 2017). ROS mediates cell death by affecting mitochondria (Zorov et al., 2014), among them, ROS-mediated apoptosis has been well studied. Due to less research, the relationship between ROS and pyroptosis has not been fully reported. Recently, there have been research reported that pyroptosis was closely related to the ROS level (Dong et al., 2020), which was also consistent with our results. However, whether ROS is the cause of pyroptosis remains unclear and is worthy of in-depth study.

There were several results indicated that lysosomes were related to radio-resistance, and targeted lysosome therapy can increase radiation sensitivity (Zhang et al., 2017; Wang et al., 2018). Lysosome-mediated radio-resistance was mainly related to autophagy. In the late stage of autophagy, lysosomes combine with autophagosomes to form autophagy-lysosomes and act as scavengers. Existing researches showed that autophagy plays a dual role in cancer development. In anti-tumor therapy, inducing autophagy can achieve tumor suppression. However, for resistant tumors, autophagy appears to be a protective mechanism that reduces the damage caused by radiation/drug (Han et al., 2018). CSC is usually associated with an increase in the level of autophagy. Autophagy allows CSC to maintain pluripotency and develop resistance, etc. (Nazio et al., 2019). Presently, our results showed that compared with the control, the autophagy of irradiated CSCs increased significantly, ∼1.7 folds of the control, while the autophagy of irradiated MCF-7 kept unchanged (Figure 5A). Studies have identified a pathway for autophagy regulation by synchronizing ATR checkpoints and double-strand break pathways (Robert et al., 2011). Therefore, CSC may use autophagy to inhibit DNA damage, thereby improving survival (Rahman et al., 2020).

For different death pathways, despite some differences, there are also some cross-talking among them. Apoptosis and necrosis are the two main pathways of cell death, and their molecular mechanisms have been extensively studied. Although originally thought to constitute a mutually exclusive cellular state, recent discoveries have revealed the need for a balanced and interactive cellular environment between these two death pathways. This was also supported by experimental data, the analysis of FAS Associated death domain (FADD)/receptor interacting protein 3 (RIP3 and FLIP/RIP3 double knockout showed that there is a complex cross-regulation of apoptosis and necrosis (Dillon et al., 2012). Besides, more and more evidences showed that autophagy and apoptosis can cooperate or antagonize with each other, thereby differentially affecting the fate of cells (Nikoletopoulou et al., 2013). In general, different death pathways interact with each other to produce the final effect. For the more precise mechanisms and the corresponding upstream and downstream influencing factors, further research is needed.

As an emerging thing, laser accelerators can complete irradiation in a very short time (∼ ns) and achieve ultra-high dose rates. But at the same time, it has stability issues, and the dose and particle types between different shots cannot be completely consistent (Lundh et al., 2012; Manti et al., 2017). We also marked in the figures. In general, more experiments are needed in the future, to further clarify the killing effect and mechanism of FLASH-RT on cancer cells and the protective mechanism of FLASH-RT on normal cells.

## Data Availability Statement

The original contributions presented in the study are included in the article/Supplementary Material, further inquiries can be directed to the corresponding author/s.

## Ethics Statement

The animal study was reviewed and approved by the Peking University Institutional Animal Care and Use Committee (Approval ID: Physics-YangG-2).

## Author Contributions

GY, CL, WM, and XY conceived the research plan. GY and CL designed the biology experiments. GY, CL, JH, JQ, and XS performed the biology experiments. CL and JH analyzed the results and produced the figures. ZM, WM, and GY designed and constructed the irradiation setup. ZM, YL, ZP, DK, SX, ZL, YGao, GQ, YS, SC, ZC, YZ, and YGeng supervised by WM performed the laser acceleration and cell irradiation experiments. ZM and YL measured the proton radiation and provided the dose data. CL wrote the initial draft of the manuscript. GY wrote the final draft of the manuscript. All authors commented on the manuscript.

## Conflict of Interest

The authors declare that the research was conducted in the absence of any commercial or financial relationships that could be construed as a potential conflict of interest.

## Abbreviations

CSC: cancer stem cell
RT: radiotherapy
IL: interleukin
ROS: reactive oxygen species
FLASH-IR: FLASH irradiation
CLAPA: compact laser plasma accelerator system
FBS: fetal bovine serum
EGF: epidermal growth factor
bFGF: basic fibroblast growth factor
FWHM: full width at half maximum
TPS: Thomson parabola spectrometer
RCF: radiochromic film
PI: propidium iodide
MDC: dansylcadaverine
MOMP: mitochondrial outer membrane permeabilization
FADD: FAS Associated death domain
RIP3: receptor interacting protein 3.
