# Supplementary material for: Association of Cancer Stem Cell Radio-Resistance Under Ultra-High Dose Rate FLASH Irradiation With Lysosome-Mediated Autophagy
Source: Front Cell Dev Biol. 2021 Apr 29;9:672693. doi: 10.3389/fcell.2021.672693 (PMC8116574; doi:10.3389/fcell.2021.672693)
Supplement: Supplementary file 1 [file Data_Sheet_1.docx]

Supplementary Material

**Dose monitoring and conversion of FLASH-IR**

The ions were created by the well-known Target Normal Sheath Acceleration mechanism (TNSA) (Passoni et al., 2010), which leads to broadband energy spectra with exponentially decreasing ion numbers up to a cut-off energy. A Thomson Parabola Spectrometer (TPS) was used to measure the spetra. The parabolic traces of ions were recorded by a micro-channel plate with a phosphor screen and imaged onto a 16-bit EMCCD camera. All the generated oxygen/carbon ions, and protons with energy below 2.7 MeV were blocked by the setups in front of the cell plane. Protons more than 2.7 MeV can deposit dose in the monolayer cell. During the cell irradiation, customized radiochromic films (RCF) EBT3 (the clear polyester facing the target has been removed) were placed directly behind the Mylar film for dose verification. The RCF films were scanned with an Epson Perfection V700 scanner in transmission mode. The dose response of EBT3 was calibrated following the literature (Reinhardt et al., 2012). The spatial distribution of protons was detected by RCF film in a single laser shot, which has a uniformity of 10% RMS (root mean square).

We performed the Monte-Carlo simulations with the code FLUKA (Böhlen et al., 2014) to simulate the protons’ trajectories and the dose delivered to the monolayer cell and to the RCF. The protons’ energy spectra input in the simulation had quasi-Boltzmann distributions reflecting the experimental measurements. All the setups in the pass way of the protons, including the beam attenuators, were contained in the simulations. The ratio of the dose in the cell to that in the RCF can be obtained from the simulations. According to multiple simulation results by taking into account the shot-to-shot fluctuation of the proton spectra, the cell-to-RCF dose ratios were 2.24 ± 0.33 for attenuation with 10 μm Aluminum film and 2.18 ± 0.33 for attenuation with 15 μm Aluminum film and copper wire mesh, respectively. Thus, the dose delivered to the cells can be obtained by multiplying the cell-to-RCF ratio to the measured dose by the RCF. In this experiment, each cell sample was irradiated with a single shot (~ 6-9 Gy). The proton beam was generated within 1 ps. Considering the difference in the flight time for protons with different energy, the radiation duration was ~1.4 ns. Thus, the dose rate was estimated to be ~10^9^ Gy/s.


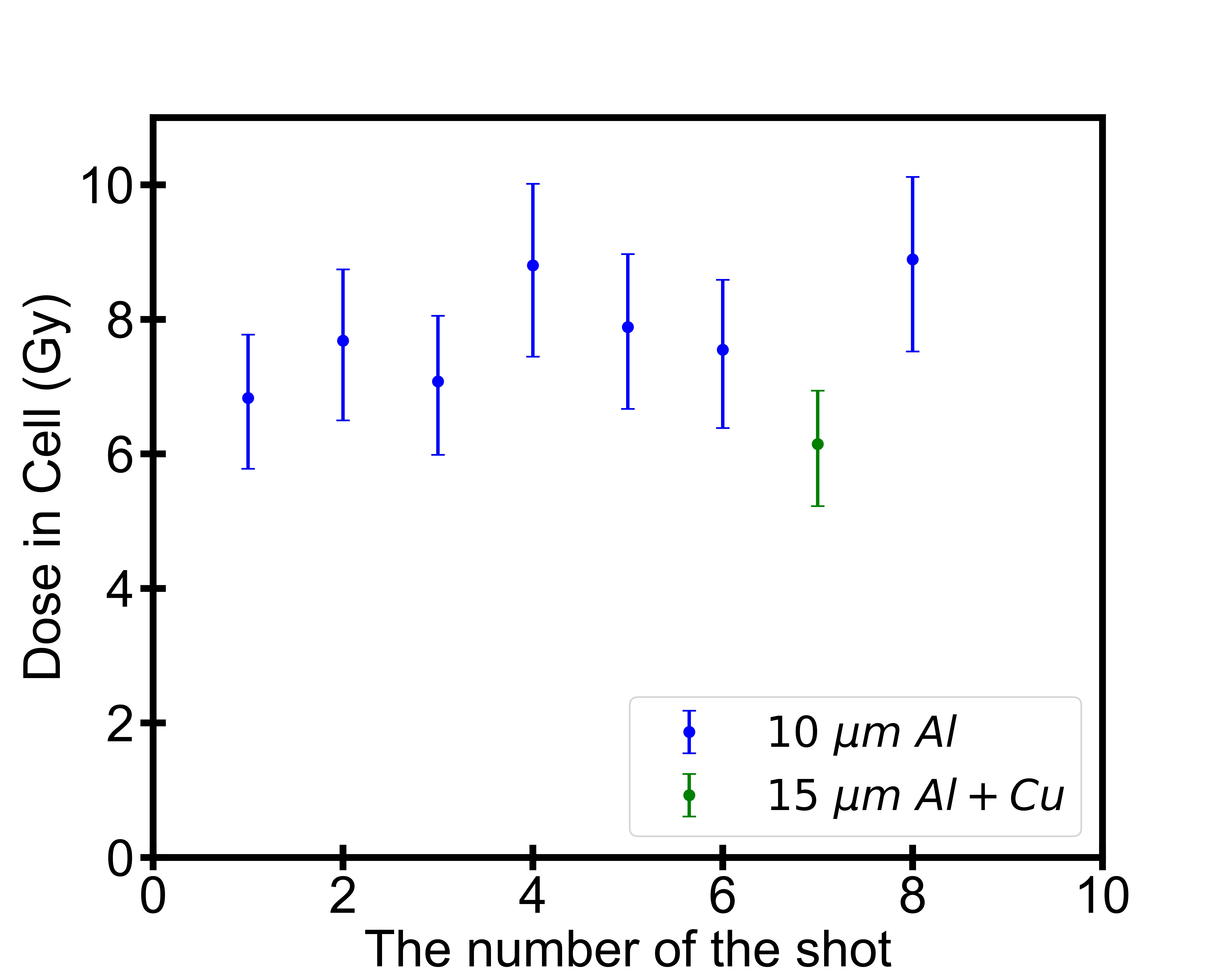


**Supplementary Figure 1**. Display of the dose of each shot. Aluminum films of different thicknesses (10 μm and 15 μm) and copper wire mesh were used in front of this Mylar film to adjust the dose.

**Supplementary Table 1**

Statistics on the size of tumors after injecting 10,000 MCF-7 cells and CSCs.

| **Time after injection of cells** | **30 days** | **31 days** | **33 days** | **34 days** | **35 days** |
| --- | --- | --- | --- | --- | --- |
| CSCs-1 | 0.520 cm | 0.565 cm | 0.855 cm | 0.965 cm | 0.980 cm |
| CSCs-2 | 0.805 cm | 0.820 cm | 0.885 cm | 0.970 cm | 1.460 cm |

**References**

Böhlen, T.T., Cerutti, F., Chin, M.P.W., Fassò, A., Ferrari, A., Ortega, P.G., et al. (2014). The FLUKA Code: Developments and Challenges for High Energy and Medical Applications. *Nuclear Data Sheets* 120**,** 211-214. doi: 10.1016/j.nds.2014.07.049.

Passoni, M., Bertagna, L., and Zani, A. (2010). Target normal sheath acceleration: theory, comparison with experiments and future perspectives. *New Journal of Physics* 12, 045012. doi: 10.1088/1367-2630/12/4/045012.

Reinhardt, S., Hillbrand, M., Wilkens, J.J., and Assmann, W. (2012). Comparison of Gafchromic EBT2 and EBT3 films for clinical photon and proton beams. *Med Phys* 39(8)**,** 5257-5262. doi: 10.1118/1.4737890.
