# Supplementary material for: Association of Cancer Stem Cell Radio-Resistance Under Ultra-High Dose Rate FLASH Irradiation With Lysosome-Mediated Autophagy
Source: Front Cell Dev Biol. 2021 Apr 29;9:672693. doi: 10.3389/fcell.2021.672693 (PMC8116574; doi:10.3389/fcell.2021.672693)
Supplement: Supplementary file 2 [file Table_1.docx]

**Supplementary Table 1**

Statistics on the size of tumors after injecting 10,000 MCF-7 cells and CSCs.

| **Time after injection of cells** | **30 days** | **31 days** | **33 days** | **34 days** | **35 days** |
| --- | --- | --- | --- | --- | --- |
| CSCs-1 | 0.520 cm | 0.565 cm | 0.855 cm | 0.965 cm | 0.980 cm |
| CSCs-2 | 0.805 cm | 0.820 cm | 0.885 cm | 0.970 cm | 1.460 cm |
