# Supplementary figures and images for: Association of Cancer Stem Cell Radio-Resistance Under Ultra-High Dose Rate FLASH Irradiation With Lysosome-Mediated Autophagy
Source: Front Cell Dev Biol. 2021 Apr 29;9:672693. doi: 10.3389/fcell.2021.672693 (PMC8116574; doi:10.3389/fcell.2021.672693)

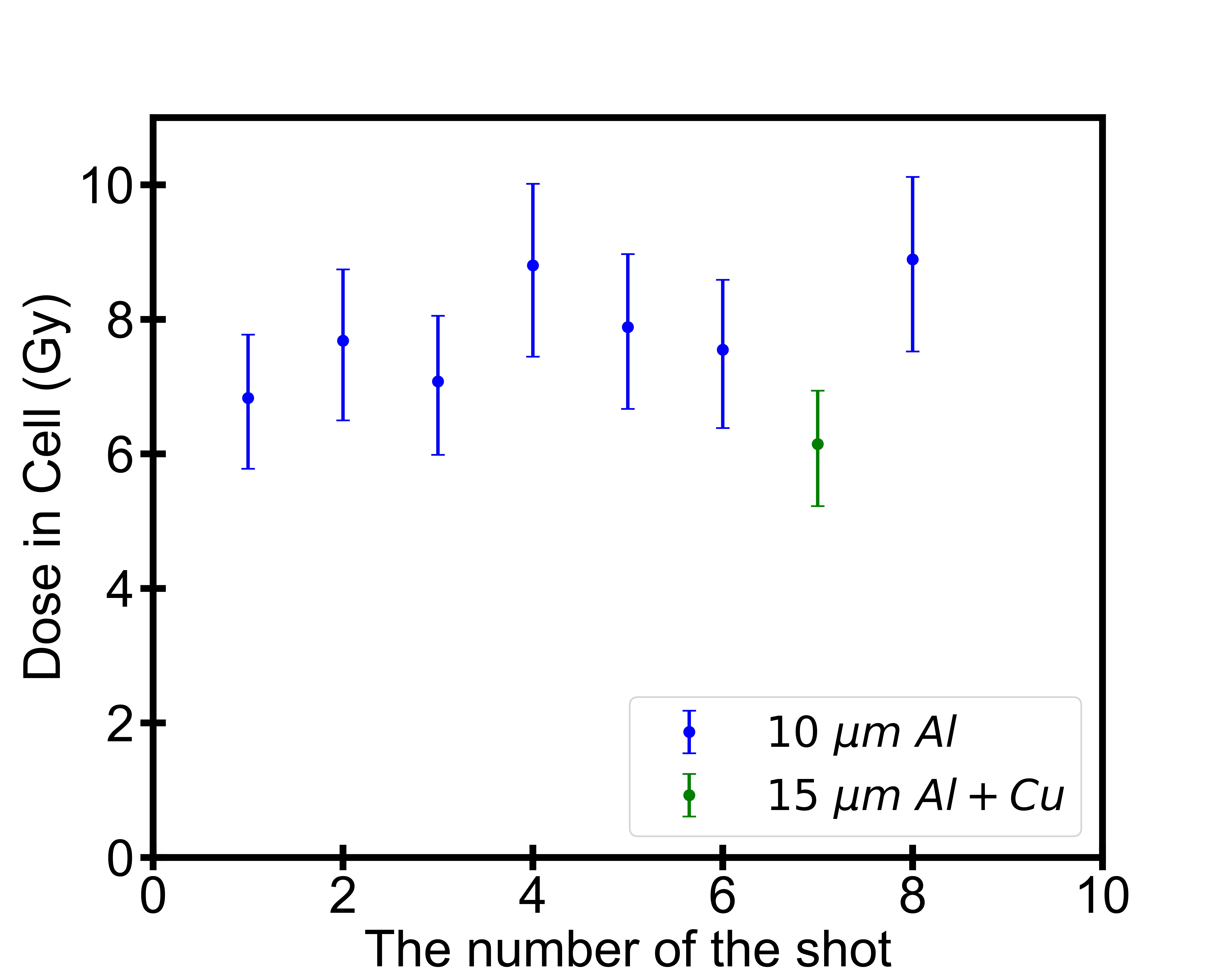

Supplement: Supplementary file 3 [file Image_1.JPEG]
